# Little Evidence of Zika Virus Infection in Wild Long-Tailed Macaques, Peninsular Malaysia

**DOI:** 10.3201/eid2502.180258

**Published:** 2019-02

**Authors:** Chong Long Chua, Yoke Fun Chan, Eva S.G. Soh Andu, Jeffrine J. Rovie-Ryan, Frankie Thomas Sitam, Khebir Verasahib, I-Ching Sam

**Affiliations:** University of Malaya, Kuala Lumpur, Malaysia (C.L. Chua, Y.F. Chan, E.S.G. Soh Andu, I-C. Sam);; Department of Wildlife and National Parks Peninsular Malaysia, Kuala Lumpur (J.J. Rovie-Ryan, F.T. Sitam);; National Public Health Laboratory, Sungai Buloh, Malaysia (K. Verasahib)

**Keywords:** Zika virus, nonhuman primates, Malaysia, *Macaca fascicularis*, macaque, neutralizing antibodies, seroprevalence, long-tailed macaques, Peninsular Malaysia, DENV-1, DENV-2, dengue virus, PRNT, FRNT, plaque reduction neutralization test, sylvatic cycle, viruses

## Abstract

We tested a sample of 234 wild long-tailed macaques (*Macaca fascicularis*) trapped in Peninsular Malaysia in 2009, 2010, and 2016 for Zika virus RNA and antibodies. None were positive for RNA, and only 1.3% were seropositive for neutralizing antibodies. Long-tailed macaques are unlikely to be reservoirs for Zika virus in Malaysia.

Zika virus, first isolated from a rhesus macaque (*Macaca mulatta*) in the Zika Forest in Uganda, reemerged in the Pacific Islands and Americas in 2015 and caused unprecedented outbreaks associated with serious congenital syndromes (*1*). The role of animal reservoirs for Zika virus is unclear, although in Africa, nonhuman primates (NHPs) are suspected to be involved in maintaining a sylvatic cycle, as they are for 2 other flaviviruses (yellow fever and dengue viruses) also transmitted by *Aedes* mosquitoes. The presence of a sylvatic cycle for Zika virus in Africa is supported by a seroprevalence of 0%–16% in African green monkeys (*Chlorocebus sabaeus*) and vervet monkeys (*Chlorocebus pygerythrus*) (*2*). However, even less is known about the potential role of NHPs in sylvatic cycles in Asia.

In Malaysia, Zika virus seropositivity has been reported in residents (*3**,**4*), monkeys (*5*), and orangutans (*4*), suggesting endemicity. Continual encroachment of human settlements into monkey habitats potentially increases human risk for exposure to monkey-associated zoonotic pathogens. We therefore evaluated Zika virus prevalence in long-tailed macaques (*M. fascicularis*), the most common macaque in Peninsular Malaysia, which is also widespread throughout Southeast Asia.

Staff of the Department of Wildlife and National Parks Peninsular Malaysia (also called Jabatan Perlindungan Hidupan Liar dan Taman Negara Semenanjung Malaysia [PERHILITAN]) traps monkeys foraging in human-populated areas and relocates them to deep forest areas (*6*). As part of PERHILITAN’s Wildlife Disease Surveillance Program, serum samples were collected from 234 long-tailed macaques trapped at >30 sites throughout Malaysia in the states of Selangor, Negeri Sembilan, Perak, Pahang, Penang, and Johor (approval no. PERHILITAN JPHL&TN(IP):100–34/1.24) and stored at −80°C. This collection comprised 145 samples acquired during October–November 2009 and October 2010 (*6*) and 89 acquired in March and August 2016, coinciding with the Zika virus global epidemics.

After extracting viral RNA from samples with a QIAamp Viral RNA Mini Kit (QIAGEN, https://www.qiagen.com), we tested samples with sufficient serum volume (n = 228) for Zika virus envelope gene by real-time PCR (*7*); none were positive. We tested all 234 samples for Zika virus neutralizing antibody by 50% plaque reduction neutralization test (PRNT_50_) on Vero cells (Appendix). In total, 6 (2.6%) samples had screening Zika virus PRNT_50_ titers ≥20 (Table); we confirmed results with a 50% Zika virus focus reduction neutralization test (FRNT_50_), and samples had titers identical to or within 1 dilution of the PRNT_50_ titer.

**Table:** Zika virus, DENV-1, and DENV-2 neutralization titers of serum samples collected from long-tailed macaques in Peninsular Malaysia, 2009, 2010, and 2016*

Sample collection period and size	No. samples†	Macaque sex and age group, ID no.	Town/city, state, coordinates	Neutralization titers			
Zika virus PRNT_50_	Zika virus FRNT_50_	DENV-1 FRNT_50_	DENV-2 FRNT_50_
October–November 2009 and October 2010, n = 145	1	Male adult, ZMW604	Bukit Serendah, Selangor, 3.36°N, 101.60°E	640	640	<20	<20
March and August 2016, n = 89	5	Female juvenile, PMW804	Manong, Perak, 4.61°N, 100.90°E	40	20	<20	<20
		Female adult, WDSP/16/009	Kuala Lipis, Pahang, 4.18°N, 102.05°E	80	80	<20	20
		Male adult, WDSP/16/006	Kuala Lipis, Pahang, 4.18°N, 102.05°E	80	80	640	160
		Male adult, WDSP/16/012	Kuala Lipis, Pahang, 4.18°N, 102.05°E	40	40	20	40
		Male adult, WDSP/16/086	Batu Pahat, Johor, 1.85°N, 102.94°E	40	20	40	20

*DENV-1, dengue virus serotype 1; DENV-2, dengue virus serotype 2; FRNT_50_, 50% focus reduction neutralization test; ID, identification; PRNT_50_, 50% plaque reduction neutralization test. †Number of samples from the first batch (n = 234) that were positive by Zika virus PRNT_50_ and further tested by FRNT_50_.

Because flavivirus antibodies are known to cross-react, these 6 samples were further examined for antibodies specific to the major known circulating flaviviruses in Malaysia, dengue virus serotypes 1 (DENV-1) and 2 (DENV-2), by FRNT_50_. A sample was considered to have evidence of Zika virus neutralizing antibody if the Zika virus PRNT_50_ titer was ≥20 and DENV-1 and DENV-2 FRNT_50_ titers were <20 (2 samples) or if the Zika virus PRNT_50_ titer was ≥20 and 4-fold greater than the DENV-1 and DENV-2 FRNT_50_ titers (1 sample). Only 3 of 6 samples fulfilled these criteria; the remaining 3 contained detectable Zika virus, DENV-1, and DENV-2 antibodies, indicating past flavivirus infection of an indeterminate type. Thus, 3 (1.3%) of 234 samples were Zika virus seropositive, although we did not test for other flaviviruses.

The 3 Zika virus–seropositive monkeys were captured 35 km away (in Bukit Serendah, Selangor), 77 km away (in Kuala Lipis, Pahang), and 164 km away (in Manong, Perak) from Bentong (Pahang), where Zika virus was first isolated outside of Africa in 1966 (*5*). Of note, 5 of 6 samples with detectable Zika virus antibodies were collected in 2016, when human Zika virus cases were occurring in Malaysia and neighboring Thailand and Singapore. The rate of Zika virus antibody detection was higher in the 2016 collection (5.6%, 5/89) than the 2009–2010 collection (0.7%, 1/145; p = 0.031 by Fisher exact test).

Our results indicate that wild long-tailed macaques in Peninsular Malaysia are exposed to Zika virus but at low levels, without evidence of viremia. This finding suggests that long-tailed macaques are unlikely involved in maintaining Zika virus sylvatic cycles in Malaysia, although the long-term dynamics of Zika virus antibodies and infection (including shedding) in macaques is unknown. This information is arguably needed before an animal can be designated a reservoir (*8*). Despite intense Zika outbreaks in humans, no active Zika virus infection and a low seroprevalence (2.9%) with low antibody titers was found in various NHP species in Brazil, suggesting that New World NHPs are unlikely to sustain sylvatic transmission cycles (*9*). Antibody responses after flavivirus infection are broadly cross-reactive and cross-neutralizing in the first few months after infection (*10*), but the effects against heterologous flaviviruses are poorly understood in wild macaques. Also, the circulation of Zika virus in macaques could be affected by the sylvatic cycles of other endemic flaviviruses. In conclusion, the low seroprevalence of Zika virus antibodies in long-tailed macaques reinforces the need to study other NHPs and mammals as reservoirs in Malaysia to elucidate Zika virus transmission and emergence.

Appendix Research methods for determining neutralizing titers against Zika and dengue viruses in serum samples from long-tailed macaques in Peninsular Malaysia, 2009, 2010, and 2016.
