# Supplementary material for: Little Evidence of Zika Virus Infection in Wild Long-Tailed Macaques, Peninsular Malaysia
Source: Emerg Infect Dis. 2019 Feb;25(2):374–6. doi: 10.3201/eid2502.180258 (PMC6346475; doi:10.3201/eid2502.180258)
Supplement: Appendix — Research methods for determining neutralizing titers against Zika and dengue viruses in serum samples from long-tailed macaques in Peninsular Malaysia, 2009, 2010, and 2016. [file 18-0258-Techapp-s1.pdf]

Article DOI: https://doi.org/10.3201/eid2502.180258

Little Evidence of Zika Virus Infection in Wild Long-Tailed Macaques, Peninsular Malaysia

Appendix

Cells and Viruses

C6/36 cells (*Aedes albopictus*, CRL-1660; American Tissue Culture Collection, https://www.atcc.org/) were maintained in Leibovitz's L-15 medium (Sigma-Aldrich, https://www.sigmaaldrich.com/) supplemented with 10% heat-inactivated fetal bovine serum (FBS; Bovagen Biologicals, https://bovogen.com/) and 8% tryptose phosphate broth (Sigma-Aldrich). Vero cells (African green monkey kidney cells, 88020401; European Collection of Authenticated Cell Cultures, https://www.phe-culturecollections.org.uk/) were maintained in Dulbecco’s Modified Eagle Medium (DMEM; Life Technologies, https://www.thermofisher.com/) in the presence of 7.5% heat-inactivated FBS, 2 mM L-glutamine (Life Technologies), and 1 mM sodium pyruvate (Hyclone, https://www.fishersci.com/). Both media contained 100 U/mL penicillin and 100 µg/mL streptomycin (Life Technologies). The C6/36 cells were incubated at 28°C in the absence of CO_2, and Vero cells were incubated at 37°C in the presence of humidity and 5% CO_2. C6/36 cells were used to propagate viruses, and Vero cells were used in virus titration and plaque reduction and focus reduction neutralization tests.

The Zika virus strain used in this study was MRS_OPY_Martinique_PaRi_2015 (European Virus Archive, https://www.european-virus-archive.com/). Dengue virus serotype 1 (DENV-1) Western Pacific and DENV-2 New Guinea C (provided by Shamala Devi Sekaran and Keivan Zandi, University Malaya, Kuala Lumpur, Malaysia) were propagated in C6/36 cells. The infectious virus supernatants were harvested after 5–7 days, precleared by centrifugation, aliquoted, and stored at –80°C. All viruses were titrated by focus immunoassay and stained with

pan flavivirus mouse monoclonal antibody D1–4G2–4-15 (4G2) (Absolute Antibody, https://absoluteantibody.com/). Plaque assays were performed to titrate Zika virus.

Virus Neutralization Assay

Plaque reduction neutralization tests (PRNTs) were performed to detect the presence of Zika virus antibodies in monkey serum samples. A rapid screen for neutralizing antibodies against Zika virus was carried out. Heat-inactivated monkey serum samples were diluted in 1× Dulbecco's phosphate-buffered saline (PBS) at a 1:20 dilution and mixed with 100–120 PFU of Zika virus prediluted in DMEM 2% FBS to a final volume of 200 µL. The virus–antibody mixture was incubated for 1 hour at 37°C before inoculation with 2.5×10^5 Vero cells in a well of a 24-well plate. The plate was further incubated for another hour at 37°C before replacing with plaque medium, 3 parts DMEM 3.5% FBS mixed with 2 parts 3% carboxymethylcellulose (Sigma-Aldrich). After 3 days incubation, the cells were fixed with 3.7% formaldehye and stained with crystal violet. The number of plaques were enumerated, and serum samples that reduced the number of plaques >75% relative to virus control were selected and retested. The neutralizing titers of serum samples were expressed as $PRNT_{50}$, the serum dilution that reduced plaque formation by 50%, and was determined with 2-fold serially diluted serum samples from 1:20 to 1:1,280.

Focus reduction neutralization tests (FRNTs) were carried out to confirm samples with Zika virus $PRNT_{50}$ titers $\geq$20 and to determine DENV-1 and DENV-2 antibody titers. A similar procedure was performed as described above, with 2-fold serially diluted serum dilutions from 1:20 to 1:1,280 mixed with 65–80 FFU of Zika virus, DENV-1, or DENV-2 except that methylcellulose (Sigma-Aldrich) was used in the plaque medium. After an incubation of 2 days (for Zika virus), 3 days (for DENV-1), and 5 days (for DENV-2), the plaque media was removed and the cells were rinsed with PBS before fixation with a chilled 1:1 mixture of methanol and acetone. The cells were blocked with 1% bovine serum albumin in PBS and incubated with 4G2 (250 ng/well) for 1 hour, followed by horseradish peroxidase–labeled goat anti-mouse IgG (Merck Millipore, http://www.emdmillipore.com/) at a 1:500 dilution for 1 hour. KPL TrueBlue Peroxidase substrate (SeraCare, https://www.seracare.com/) was used to visualize foci. Neutralizing titers were expressed as $FRNT_{50}$, the serum dilution that reduced foci formation by

50% relative to virus control in the absence of serum. ZKA185, a neutralizing human monoclonal antibody (Absolute Antibody), served as the positive control for Zika virus neutralization assays and was used at a final concentration of 10 µg/mL.
